# Early prediction of live birth for assisted reproductive technology patients: a convenient and practical prediction model

**DOI:** 10.1038/s41598-020-79308-9

**Published:** 2021-01-11

**Authors:** Hong Gao, Dong-e Liu, Yumei Li, Xinrui Wu, Hongzhuan Tan

**Affiliations:** 1 School of Nursing, University of South China, Hengyang, 421001 Hunan China; 2 Department of Epidemiology and Health Statistics, XiangYa School of Public Health, Central South University, Changsha, 410008 Hunan China; 3 Reproductive Medicine Centre, Xiangya Hospital of Central South University, Changsha, 410008 Hunan China

**Keywords:** Reproductive disorders, Epidemiology, Outcomes research

## Abstract

Live birth is the most important concern for assisted reproductive technology (ART) patients. Therefore, in the medical reproductive centre, obstetricians often need to answer the following question: “What are the chances that I will have a healthy baby after ART treatment?” To date, our obstetricians have no reference on which to base the answer to this question. Our research aimed to solve this problem by establishing prediction models of live birth for ART patients. Between January 1, 2010, and May 1, 2017, we conducted a retrospective cohort study of women undergoing ART treatment at the Reproductive Medicine Centre, Xiangya Hospital of Central South University, Hunan, China. The birth of at least one live-born baby per initiated cycle or embryo transfer procedure was defined as a live birth, and all other pregnancy outcomes were classified as no live birth. A live birth prediction model was established by stepwise multivariate logistic regression. All eligible subjects were randomly allocated to two groups: group 1 (80% of subjects) for the establishment of the prediction models and group 2 (20% of subjects) for the validation of the established prediction models. The sensitivity, specificity, positive predictive value (PPV), and negative predictive value (NPV) of each prediction model at different cut-off values were calculated. The prediction model of live birth included nine variables. The area under the ROC curve was 0.743 in the validation group. The sensitivity, specificity, PPV, and NPV of the established model ranged from 97.9–24.8%, 7.2–96.3%, 44.8–83.8% and 81.7–62.5%, respectively, at different cut-off values. A stable, reliable, convenient, and satisfactory prediction model for live birth by ART patients was established and validated, and this model could be a useful tool for obstetricians to predict the live rate of ART patients. Meanwhile, it is also a reference for obstetricians to create good conditions for infertility patients in preparation for pregnancy.

## Introduction

Live birth is the most important concern for assisted reproductive technology (ART) patients; additionally, it is the only criterion used to determine whether ART treatment is successful. In recent years, ART in humans has developed very quickly, but the live birth rate is still less than optimal^1^. Fundamentally, numerous key factors play important roles in live birth outcomes, including embryo parameters^2,3^, reproductive hormone levels^4–6^, and the patient’s age^7,8^, among others; however, the degrees to which these factors affect birth outcomes are not clear. Consequently, it is very significant to investigate how these factors affect live birth outcomes, determine their degrees of influence and then establish live birth prediction models.

Previous studies have used various strategies to predict live birth for different sub-groups of ART patient, including in vitro fertilization (IVF) patients^9^, intracytoplasmic sperm injection (ICSI) patients^10^, ICSI patients with uncompromised ovarian reserve^11^, and poor ovarian responders^12,13^. Different strategies have included risk scoring systems^14^ deep phenotyping^9^, granulosa cell biomarkers^1^, and clinical characteristics (female age, testicular sperm extraction cycle, male and female reproductive hormones, spermatozoa parameters, infertility diagnosis, and oocyte parameters)^10,11,15^. The sensitivity and specificity of these live birth prediction models exhibit large variations. Prajna Banerjee et al.^9^ used 52 clinical characteristics to establish a model. The area under the receiver operating characteristic (ROC) curve was up to 0.80 for IVF patients in their first cycles, but in their subsequent treatments, this value decreased to less than 0.68. Although many live birth prediction models have been established, they are rarely used in clinical practice. The main reasons may include the following: (1) they cannot be applied to all ART patients because the model is based on only 1–2 types of ART patients; (2) some predictors need more complicated and expensive laboratory tests; (3) the use of these models is not sufficiently convenient, and (4) some models are less accurate than others for predicting live birth.

We aimed to establish a convenient and practical live birth prediction model that has higher predictive value and that can be applied to all ART patients.

## Results

There were 15,717 ART treatments performed from 2012 to 2017. Of these, 1891 subjects who had missing information on their live birth outcomes were excluded, leaving 13,826 subjects for analysis. Among them, 80% of the subjects (11,071) were allocated to group 1 (establishment model), and 20% of them (2755) were allocated to group 2 (validation model).

### Univariate analysis results

We analysed the relationships between the variables and live birth outcomes by univariate analysis. Twenty-two variables were found to be significantly associated with live birth outcomes (*p*-value < 0.05, Tables 1, 2).

**Table 1:** Comparison of the characteristics between the live birth and no live birth groups comprising 13,826 ART patients from 2010–2017 (continuous variables). (Mean ± SD)**.**

Variables	Live birth n = 6012	No live birth n = 7814	*p-*value
**Pre-ART factors**			
Maternal age, year	30.63 ± 4.36	32.67 ± 5.50	< 0.001
Body mass index, kg/m^2^	21.61 ± 2.97	21.97 ± 3.05	< 0.001
Uterine volume, mL	52.21 ± 22.37	54.42 ± 25.83	< 0.001
Female infertility duration, year	4.67 ± 3.31	5.42 ± 3.98	< 0.001
No. of previous pregnancies	1.07 ± 1.32	1.27 ± 1.46	< 0.001
No. of abortions	0.62 ± 0.97	0.78 ± 1.13	< 0.001
Basal FSH^a^, mIU/mL	6.79 ± 18.60	7.28 ± 7.49	< 0.001
Number of previous ART treatments	1.72 ± 0.99	2.01 ± 1.18	< 0.001
**Protocol and treatment factors**			
Total dose of gonadotropin, IU	2007.61 ± 841.34	2049.03 ± 1074.933	< 0.001
No. of antral follicles	6.25 ± 3.29	5.50 ± 3.33	< 0.001
Total no. of oocytes	11.01 ± 4.50	9.12 ± 4.66	< 0.001
Sperm concentration, million/mL	109.90 ± 98.18	104.73 ± 92.83	0.04
Sperm viability, %	41.28 ± 22.31	41.38 ± 22.14	0.87
Sperm progressive motility, %	34.82 ± 23.33	34.87 ± 23.54	0.68
Endometrial thickness before embryo transfer, mm	10.51 ± 2.10	9.99 ± 2.11	< 0.001
Total no. of transferred embryos	1.98 ± 0.27	1.88 ± 0.45	< 0.001

^a^FSH, follicle-stimulating hormone.

**Table 2:** Comparison of the live birth rates of the different sub-groups of the 13,826 ART patients from 2010–2017 (categorical variables).

Variables	N	Live birth (n)	Live birth rate (%)	*p*-value
**Pre-ART factors**				
Maternal education				< 0.001
Under 6 years	1086	392	36.1	
6–9 years	6186	2723	44.0	
10–12 years	1737	834	48.0	
13 years and over	4341	2047	47.2	
Infertility diagnosis				< 0.001
Male factors	1890	818	43.3	
Ovulation dysfunction	58	25	43.1	
Decreased ovarian reserve	150	36	24.0	
Tubal factors	9568	4164	43.5	
Uterine factors	142	54	38.0	
Chromosome abnormality	14	5	35.7	
Unexplained factors	44	15	34.1	
Male + female factors	1700	774	45.5	
**Protocol and treatment factors**				
Stimulation protocol				< 0.001
Long protocol	5648	3024	53.5	
Short protocol	1752	521	29.7	
Other protocol	2703	752	27.8	
Artificial insemination technology				0.02
IVF	9820	4344	44.2	
ICSI	2871	1215	42.3	
IVF + ICSI	884	358	40.5	
IUI^a^	77	27	35.1	
Type of embryo transfer				< 0.001
Fresh embryo	7431	3544	47.7	
Frozen-thawed embryo	6309	2442	38.7	
Quality of the transferred embryos^b^				< 0.001
I	11,585	5482	47.3	
II	1112	334	30.0	
III	443	62	14.0	

^a^IUI, intrauterine insemination.

^b^Quality of the transferred embryos: I is the best-quality embryo, followed by II and III.

### Logistic regression analysis and prediction model establishment

Based on our univariate analysis results, we found that maternal age, body mass index, number of previous ART treatments, female infertility duration, number of previous pregnancies, number of abortions, basal FSH, sperm concentration, endometrial thickness before embryo transfer, number of antral follicles, total number of oocytes, sperm viability, sperm progressive motility, type of embryo transfer, quality of transferred embryos, maternal education, infertility diagnosis, uterine volume, artificial insemination technology used, stimulation protocol, total number of transferred embryos, and total dose of gonadotropin were significantly associated with live birth. We used these variables as independent variables to perform multiple logistic regression analysis. The variables are presented in Table 3. We found there was not multicollinearity among those variables (Tol > 0.1, VIF < 10, shown in Table 4). Likelihood ratio forward stepwise method (α = 0.05 for entry, and α = 0.10 for removal) was used in the logistic regression. Finally, the prediction model of live birth was established, including nine variables (shown in Table 4). Each step of this model is shown in Supplementary Table S1. The area under the ROC curve was 0.722 (95% CI, 0.709–0.735). The model is as follows: Logit P =  − 1.857 + 0.199X_1_ + 0.150X_2_ + 0.276X_3_ + 0.077X_5_ − 0.149X_8_ + 1.205X_9_ + 0.690X_12_ + 0.770X_13_ + 0.534X_19._

**Table 3:** Variable assignment in the multivariate logistic regression analysis.

Variables	Variable names	Assignment statement
Maternal age (year)	X_1_	1 = < 25, 2 = 25~, 3 = 30~, 4 = ≥ 35
Maternal education (year)^a^	X_2_	1 = < 10, 0 = ≥ 10
Number of previous ART treatments	X_3_	1 = 1, 2 = 2, 3 = ≥ 3
Uterine volume (mL)^a^	X_4_	1 = < 30, 0 = 30~, 2 = 50~, 3 = ≥ 70
No. of abortions	X_5_	1 = 0, 2 = 1, 3 = 2, 4 = ≥ 3
Sperm concentration (million/mL)	X_6_	1 = < 43.00, 2 = 43.00~, 3 = 85.00 ~ , 4 = ≥ 144.40
Infertility diagnosis^a^	X_7_	1 = male factors, 2 = ovulation dysfunction, 3 = decreased ovarian reserve, 4 = tubal factors, 5 = uterine factors, 6 = chromosome abnormality, 7 = unexplained, 0 = male + female factors
Endometrial thickness before embryo transfer (mm)	X_8_	1 = < 8, 2 = 8 ~, 3 = 10 ~ , 4 = ≥ 12
Total no. of transferred embryos^a^	X_9_	1 = 1, 0 = ≥ 2
Total dose of gonadotropin (IU)^a^	X_10_	1 = < 1500, 0 = 1500 ~ , 2 = 2000 ~ , 3 = ≥ 2500
Total no. of oocytes	X_11_	1 = < 8, 2 = 8 ~ , 3 = ≥ 13
Quality of the transferred embryos	X_12_	1 = I, 2 = II, 3 = III
Stimulation protocol^a^	X_13_	0 = long protocol, 1 = short protocol, 1 = other protocol
Sperm viability (%)	X_14_	0 = < 40.00, 1 = ≥ 40.00
Sperm progressive motility (%)	X_15_	0 = < 32.00, 1 = ≥ 32.00
BMI (kg/m^2^)	X_16_	1 = < 18.50, 2 = 18.50 ~ , 3 = 24.00~, 4 = ≥ 28.00
Female infertility duration (yr)	X_17_	1 = < 3, 2 = 3 ~ , 3 = 5 ~ , 4 = ≥ 7
No. of previous pregnancies	X_18_	1 = 0, 2 = 1, 3 = 2, 4 = 3, 5 = ≥ 4
Basal FSH (mIU/mL)	X_19_	0 = < 10, 1 = ≥ 10
No. of antral follicles	X_20_	0 = < 5, 1 = ≥ 5
Type of embryo transfer	X_21_	0 = fresh embryo, 1 = frozen-thawed embryo
Artificial insemination technology^a^	X_22_	0 = IVF, 1 = ICSI, 2 = IVF + ICSI, 3 = IUI
Live birth outcome	Y	1 = no live birth, 0 = live birth

^a^X_2_, X_4_, X_7_, X_9_, X_10_, X_13_, X_22_ were entered into the models as dummy variables, and the group with the highest live birth rate was selected as the reference group, which was assigned “0”.

**Table 4:** The association between live birth and the predictive variables included in the logistic predicting model.

Variables	B_0_	b_i_	*p*	Collinearity Statistics	
				Tolerance	VIF
Maternal age (X1)	0.199	0.035	< 0.001	0.879	1.138
Maternal education (X2)	0.150	0.058	0.009	0.996	1.004
Number of previous ART treatments (X3)	0.276	0.047	< 0.001	0.679	1.472
No. of abortions (X5)	0.077	0.034	0.023	0.905	1.105
Endometrial thickness before embryo transfer (X8)	− 0.149	0.033	< 0.001	0.881	1.135
Total no. of transferred embryos (X9)	1.205	0.119	< 0.001	0.949	1.053
Quality of the transferred embryos (X12)	0.690	0.071	< 0.001	0.983	1.018
Stimulation protocol (X13)	0.770	0.071	< 0.001	0.654	1.530
Basal FSH (X19)	0.534	0.123	< 0.001	0.954	1.048
Constant	− 1.857	0.175	< 0.001		

### Verification prediction model

**Figure 1:**
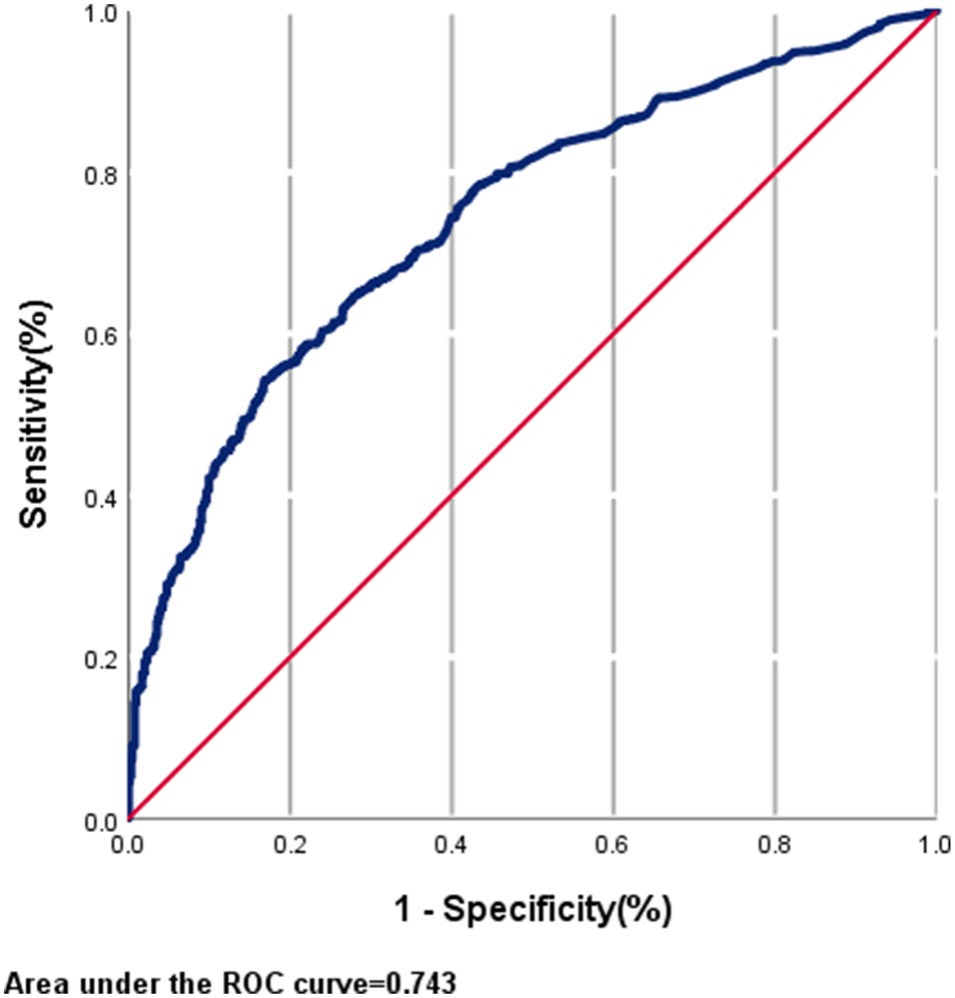
Receiver operating characteristic (ROC) curve of the live birth prediction model for ART patients.

We performed a validation of the prediction model of live birth by using the model in the validation group [with 20% of the subjects (2755)]. The area under the ROC curve was 0.743 (95% CI, 0.719–0.768) (Fig. 1). Table 5 shows the practical predictive value for live birth with different cut-off values. The sensitivity(SE) and specificity(SP) ranged from 97.9–24.8% and 7.2–96.3%, respectively, at different cut-off values.

**Table 5:** The validity of the prediction model with different cut-off values in different groups (%).

Cut-off value	Group 1				Group 2			
	SE	SP	PPVa	NPVb	SE	SP	PPV	NPV
0.25	99.7	1.7	43.9	88.0	97.9	7.2	44.8	81.7
0.30	95.5	14.3	46.2	80.5	93.8	20.2	47.5	80.9
0.35	86.7	35.1	50.7	77.4	89.0	34.7	51.2	80.4
0.40	78.7	0.50	54.8	75.3	84.2	43.9	53.6	78.3
0.45	72.4	58.9	57.6	73.5	77.6	57.3	58.3	76.9
0.50	67.8	66.3	60.8	72.8	68.3	65.9	60.7	73.0
0.55	62.2	72.5	63.5	71.4	65.1	71.3	63.6	72.6
0.60	55.3	78.9	66.9	69.6	57.5	78.7	67.5	70.6
0.65	44.5	84.9	69.4	66.5	50.3	84.7	71.7	68.9
0.70	34.4	90.8	74.2	64.3	43.1	89.4	75.8	67.1
0.75	25.4	94.3	77.4	62.1	32.4	93.4	79.1	64.2
0.80	18.3	96.5	80.1	60.5	24.8	96.3	83.8	62.5

^a^PPV: positive predictive value.

^b^NPV: negative predictive value.

## Discussion

We established a prediction model of live birth by multivariable logistic regression analysis, and this model included 9 common variables. In the establishment model group, the ROC value was 0.722, and there was good calibration. In the validation model group set, the ROC value was 0.743, and the model was calibrated well.

Our model has several obvious advantages. Our model is a convenient and practical prediction model because information on all the variables included in the model is generally available in the clinic, and there is no need for any special test. Some predictors in our model, such as endometrial thickness, stimulation protocol, and embryo parameters, can be the focus of interventions. Therefore, the model has a certain predictive value and instructional clinical value in early treatments. The model can predict a live birth in the early pregnancy stage because information on almost all the variables included in the model is available at the beginning of pregnancy. Moreover, our model has acceptable clinical predictive value, and the area under the ROC curve reached 0.743 in the validation group, which is higher than the values of most of the previously reported models that were similar to our model^10,13,16^^.^ Although the ROC values of some models are larger than ours, the variables in these models, such as gene or granulosa cell biomarkers, may be inconvenient to assess^1,17^. Moreover, a variable, such as gene expression, may be unchangeable and have no preventive value^1^. Furthermore, information on variables, such as HCG and progesterone may only be attainable after pregnancy is achieved and cannot be used for the prediction of a live birth^9,18,19^. Many previous prediction models of live birth are not applicable to all ART patients but are instead only applicable for a specific infertility subgroup^10,20–22^; however, our model is suitable for different artificial insemination technologies and all ART patients.

We have established a highly discriminatory, well-calibrated, robust, and practical prediction model that can use available clinical data to predict the live birth rate and may be transferred to corresponding computer software for easy operation. Clinicians and public health workers can easily use this model to identify high-risk populations for the management by ART.

As we all know, breeding a new life is a very complex process, which will be affected by many known and unknown factors. Especially, for infertility patients, the condition will be more complex and changeable. Medical technology level of different hospitals and doctors quite naturally plays a fundamental role. To date, it is hard to predict live birth rates before embryo transfer. In the early stage of infertility treatment, patients and doctors are most concerned about “How many normal oocytes are there?”, “How many normal sperm are there?” and “How many embryos can be transferred?” Therefore, the prediction of live birth rate is at least based on the successful embryo transfer. However, this finding can be used as a guidance to try to create good conditions for infertility patients in preparation for pregnancy.

Our live birth prediction models were further validated with a separate sample, allowing us the ability to evaluate the true predictive performance of the models when they are being used in other populations. We also examined the impacts of different cut-off values on sensitivity, specificity, PPV and NPV, to establish an appropriate reference range. Clinicians and public health workers could conveniently select different cut-off values in their live birth assessment process to obtain optional results.

There are several limitations to this study. Our data were obtained only from a large reproductive medicine centre, and the ROC values of our model are not the largest among the reported models. Therefore, we do not think the model is unchangeable. With the development of medical technology, new variables will be added to our model, and our prediction model will be continuously optimized. In addition, the applicability of the model in other clinics needs to be further verified, which will be our next research work.

In conclusion, a prediction model for live birth by ART patients was established and validated. The model is stable, reliable, convenient, and satisfactory; furthermore, this model could be a useful tool for early-gestation predictions by obstetricians of whether or not a ART patient has a high probability of a live birth and for trying to create good conditions for ART patients in preparation for pregnancy.

## Methods

### Study data

Between January 1 2010 and May 1 2017, we conducted a retrospective cohort study of women undergoing ART treatments at the Reproductive Medicine Centre, Xiangya Hospital of Central South University, Hunan, China. All data of the subjects were retrieved from the electronic medical records (Haitai, Nanjing, China) of Xiangya Hospital Central South University. The inclusion criteria for data analysis were as follows: (1) completion of the basic medical examination; (2) completion of the entire ART treatment cycle; (3) complete record of the couple’s basic information; and (4) thorough documentation of the live birth outcome.

### Outcome measures

In our study, we focused on live birth. The birth of at least one live-born baby per initiated cycle or embryo transfer procedure was defined as a live birth, and all the other adverse pregnancy outcomes were classified as no live birth.

### Statistical analysis

A live birth prediction model was established by stepwise multivariate logistic regression (α = 0.05 for entry, and α = 0.10 for removal). When establishing the model, the criteria for selecting predictive variables were as follows: first, *p* value was less than 0.05; second, it contributed to improving the area under the ROC curve. All eligible subjects were randomly allocated to two groups: group 1 (80% of subjects) for the establishment of the prediction models and group 2 (20% of subjects) for the validation of the established prediction models. Subjects of the two groups were independent without repetition. The areas under the ROC curve generated by the logistic regression model were applied to evaluate the effectiveness of the prediction models. We further calculated the sensitivity, specificity, positive predictive value, and negative predictive value of the prediction models with different cut-off values.

All data were managed and analysed using the statistical package for social sciences (SPSS) software version 17.0 (Chicago, IL, SPSS Inc. 2008) and Excel (Microsoft Corp., Redmond, WA, USA). Measurement data are described as the mean ± standard deviation (SD), and enumeration data are described as numbers (percentages). All *p* values corresponded to two-sided tests, and *p* < 0.05 was considered statistically significant.

### Ethical approval and consent to participate

The study was approved by the Ethics Committee of Xiangya Hospital of Central South University, mainly including the use of data from various clinical examination and laboratory tests of patients. All infertility patients presenting to the Reproductive Medicine Centre, Xiangya Hospital of Central South University, Hunan, China, who were planned for ART treatments and who signed the informed consent were enrolled in the study from January 1 2010 and May 1 2017. In addition, we confirmed that all methods were performed in accordance with the assisted reproductive technology relevant guidelines and regulations.

## Supplementary Information

Supplementary Table.

## Data Availability

The datasets used and/or analysed during the current study are available from the corresponding author on reasonable request.

## References

[1] Kordus RJ, LaVoie HA (2017). Granulosa cell biomarkers to predict pregnancy in ART: pieces to solve the puzzle. Reproduction.

[2] Zhao YY, Yu Y, Zhang XW (2018). Overall blastocyst quality, trophectoderm grade, and inner cell mass grade predict pregnancy outcome in euploid blastocyst transfer cycles. Chin. Med. J. (Engl.).

[3] Aparicio-Ruiz B, Romany L, Meseguer M (2018). Selection of preimplantation embryos using time-lapse microscopy in in vitro fertilization: state of the technology and future directions. Birth Defects Res..

[4] Zhao W (2018). Effects of oestradiol for luteal phase support in fresh embryo transfer cycles: a retrospective cohort study. Clin. Endocrinol. (Oxf.).

[5] Seikkula J (2018). Mid-luteal phase gonadotropin-releasing hormone agonist support in frozen-thawed embryo transfers during artificial cycles: a prospective interventional pilot study. J. Gynecol. Obstet. Hum. Reprod..

[6] Daney DMF (2017). What are the likely IVF/ICSI outcomes if there is a discrepancy between serum AMH and FSH levels? A multicenter retrospective study. J. Gynecol. Obstet. Hum. Reprod..

[7] Amsiejiene A (2017). The influence of age, body mass index, waist-to-hip ratio and anti-Mullerian hormone level on clinical pregnancy rates in ART. Gynecol. Endocrinol..

[8] Srouji SS (2005). Predicting in vitro fertilization live birth using stimulation day 6 estradiol, age, and follicle-stimulating hormone. Fertil. Steril..

[9] Banerjee P (2010). Deep phenotyping to predict live birth outcomes in in vitro fertilization. Proc. Natl. Acad. Sci. USA.

[10] Meijerink AM (2016). Prediction model for live birth in ICSI using testicular extracted sperm. Hum. Reprod..

[11] Goldman RH (2017). Predicting the likelihood of live birth for elective oocyte cryopreservation: a counseling tool for physicians and patients. Hum. Reprod..

[12] Lainas TG (2015). Live birth rates after modified natural cycle compared with high-dose FSH stimulation using GnRH antagonists in poor responders. Hum. Reprod..

[13] Lehert P (2018). Predicting live birth for poor ovarian responders: the PROsPeR concept. Reprod. Biomed. Online.

[14] Michailidou-Ahmed C, Sharpe AA, Burrell EV, Blower JA, Potdar N (2016). HBA score in relation to donor semen profiles and live birth rates: a preliminary study. Hum. Fertil. (Camb.).

[15] Peng J, Zhang Z, Yuan Y, Cui W, Song W (2017). Pregnancy and live birth rates after microsurgical vasoepididymostomy for azoospermic patients with epididymal obstruction. Hum. Reprod..

[16] Dhillon RK (2016). Predicting the chance of live birth for women undergoing IVF: a novel pretreatment counselling tool. Hum. Reprod..

[17] Bracewell-Milnes T (2017). Metabolomics as a tool to identify biomarkers to predict and improve outcomes in reproductive medicine: a systematic review. Hum. Reprod. Update.

[18] Blakemore JK, Kofinas JD, McCulloh DH, Grifo J (2017). Serum progesterone trend after day of transfer predicts live birth in fresh IVF cycles. J. Assist. Reprod. Genet..

[19] Iliodromiti S, Kelsey TW, Wu O, Anderson RA, Nelson SM (2014). The predictive accuracy of anti-Mullerian hormone for live birth after assisted conception: a systematic review and meta-analysis of the literature. Hum. Reprod. Update.

[20] van Loendersloot LL, van Wely M, Repping S, Bossuyt PM, van der Veen F (2013). Individualized decision-making in IVF: calculating the chances of pregnancy. Hum. Reprod..

[21] Lintsen AM (2007). Predicting ongoing pregnancy chances after IVF and ICSI: a national prospective study. Hum. Reprod..

[22] Alson S, Bungum LJ, Giwercman A, Henic E (2018). Anti-mullerian hormone levels are associated with live birth rates in ART, but the predictive ability of anti-mullerian hormone is modest. Eur. J. Obstet. Gynecol. Reprod. Biol..

