# Supplementary material for: Early prediction of live birth for assisted reproductive technology patients: a convenient and practical prediction model
Source: Sci Rep. 2021 Jan 11;11:331. doi: 10.1038/s41598-020-79308-9 (PMC7801433; doi:10.1038/s41598-020-79308-9)
Supplement: Supplementary file 1 — Supplementary Table. [file 41598_2020_79308_MOESM1_ESM.docx]

**Supplementary Table S1** The specific steps of establishing the model by forward stepwise regression.

| Regression steps | Variables | B_0_ | b_i_ | *P* | ROC |
| --- | --- | --- | --- | --- | --- |
| Step 1 | Stimulation protocol | 1.218 | 0.058 | <0.001 | 0.626 |
| Step 2 | Stimulation protocol | 1.126 | 0.059 | <0.001 | 0.656 |
|  | Total no. of transferred embryos | 1.316 | 0.116 | <0.001 |  |
| Step 3 | Stimulation protocol | 1.139 | 0.059 | <0.001 | 0.683 |
|  | Quality of the transferred embryos | 0.744 | 0.070 | <0.001 |  |
|  | Total no. of transferred embryos | 1.281 | 0.118 | <0.001 |  |
| Step 4 | Stimulation protocol | 1.087 | 0.060 | <0.001 | 0.700 |
|  | Quality of the transferred embryos | 0.708 | 0.071 | <0.001 |  |
|  | Total no. of transferred embryos | 1.249 | 0.118 | <0.001 |  |
|  | Maternal age | 0.250 | 0.033 | <0.001 |  |
| Step 5 | Stimulation protocol | 0.856 | 0.070 | <0.001 | 0.703 |
|  | Quality of the transferred embryos | 0.698 | 0.071 | <0.001 |  |
|  | Total no. of transferred embryos | 1.232 | 0.118 | <0.001 |  |
|  | Number of previous ART treatments | 0.287 | 0.046 | <0.001 |  |
|  | Maternal age | 0.238 | 0.033 | <0.001 |  |
| Step 6 | Stimulation protocol | 0.795 | 0.071 | <0.001 | 0.704 |
|  | Quality of the transferred embryos | 0.705 | 0.071 | <0.001 |  |
|  | Total no. of transferred embryos | 1.215 | 0.119 | <0.001 |  |
|  | Number of previous ART treatments | 0.262 | 0.046 | <0.001 |  |
|  | Maternal age | 0.239 | 0.033 | <0.001 |  |
|  | Endometrial thickness before embryo transfer | -0.158 | 0.032 | <0.001 |  |
| Step 7 | Stimulation protocol | 0.768 | 0.071 | <0.001 | 0.709 |
|  | Quality of the transferred embryos | 0.691 | 0.071 | <0.001 |  |
|  | Total no. of transferred embryos | 1.192 | 0.119 | <0.001 |  |
|  | Number of previous ART treatments | 0.266 | 0.046 | <0.001 |  |
|  | Maternal age | 0.223 | 0.033 | <0.001 |  |
|  | Endometrial thickness before embryo transfer | -0.160 | 0.032 | <0.001 |  |
|  | Basal FSH | 0.535 | 0.123 | <0.001 |  |
| Step 8 | Stimulation protocol | 0.768 | 0.071 | <0.001 | 0.721 |
|  | Quality of the transferred embryos | 0.690 | 0.071 | <0.001 |  |
|  | Total no. of transferred embryos | 1.202 | 0.119 | <0.001 |  |
|  | Number of previous ART treatments | 0.268 | 0.046 | <0.001 |  |
|  | Maternal age | 0.219 | 0.033 | <0.001 |  |
|  | Endometrial thickness before embryo transfer | -0.159 | 0.032 | <0.001 |  |
|  | Maternal education | 0.148 | 0.058 | 0.010 |  |
|  | Basal FSH | 0.535 | 0.123 | <0.001 |  |
| Step 9 | Stimulation protocol | 0.770 | 0.071 | <0.001 | 0.722 |
|  | Quality of the transferred embryos | 0.690 | 0.071 | <0.001 |  |
|  | Total no. of transferred embryos | 1.205 | 0.119 | <0.001 |  |
|  | Number of previous ART treatments | 0.276 | 0.047 | <0.001 |  |
|  | Maternal age | 0.199 | 0.035 | <0.001 |  |
|  | Endometrial thickness before embryo transfer | -0.149 | 0.033 | <0.001 |  |
|  | No. of abortions | 0.077 | 0.034 | 0.023 |  |
|  | Maternal education | 0.150 | 0.058 | 0.009 |  |
|  | Basal FSH | 0.534 | 0.123 | <0.001 |  |
